# Factors associated with diabetic foot among type 2 diabetes in Northern area of Saudi Arabia: a descriptive study

**DOI:** 10.1186/s13104-019-4088-4

**Published:** 2019-01-22

**Authors:** Manal S. Fawzy, Mariam A. Alshammari, Ashwaq A. Alruwaili, Rehab T. R. Alanazi, Jewaher A. M. Alharbi, Abdulaziz Mohammed R. Almasoud, Reem A. Alshammari, Eman A. Toraih

**Affiliations:** 1 Department of Biochemistry, Faculty of Medicine, Northern Border University, P.O. Box: 1321, Arar, Saudi Arabia; 2 Faculty of Medicine, Northern Border University, Arar, Saudi Arabia; 3 Faculty of Pharmacy, Northern Border University, Rafha, Saudi Arabia; 4 Rafha Central Hospital, Rafha, Saudi Arabia; 5 Department of Medical Biochemistry and Molecular Biology, Faculty of Medicine, Suez Canal University, Ismailia, Egypt; 6 Genetics Unit, Department of Histology and Cell Biology, Faculty of Medicine, Suez Canal University, Ismailia, Egypt

**Keywords:** Diabetic foot, Risk factors, Northern Borders area, Saudi Arabia

## Abstract

**Objective:**

Foot complications are considered to be a devastating consequence of type 2 diabetes mellitus (T2DM), posing a major medical and economic burden. A prospective study was conducted at researchers’ area “Northern area of Saudi Arabia” to determine the factors associated with diabetic foot (DF) among T2DM patients. Identifying the extent of this problem and the associated factors will enable the health providers to imply early preventive measurements.

**Results:**

Two hundred T2DM patients with/without DF (n = 100 for each group) were recruited. In total, the mean (SD) age of participants was 56 (± 12.2) years and nearly 70% of the patients were females. They showed a trend for higher frequency of impaired vibration perception, light touch pressure, proprioception and pain sensation than males in T2DM with DF. In univariate analysis, older age, long duration of diabetes and poor glycemic control reflected in high levels of HbA1c were significant factors associated with DF (OR = 4.1, 95% CI 2.3–7.4, *P *< 0.0001; OR = 6.5, 95% CI (4.9–9.3), *P *< 0.0001, and OR = 1.1, 95% CI (1.05–1.3), *P *= 0.002, respectively). Taken together, the current results could highlight the importance of epidemiological studies to raise the awareness of this important health care problem around the country.

## Introduction

Saudi Arabia is one of the top ten countries (the 6th rank) for diabetes prevalence and it is speculated to continue with a prevalence rate of 20.0% in the age group 20–79 years in the coming two decades [1]. The diabetic foot is characterized by a classical triad of neuropathy, ischemia, and infection and no wonder is one of the most serious complications of diabetes [2]. Diabetic foot ulcers are common and estimated to affect 15% of all diabetic individuals during their lifetime and is now appreciated that 15–20% of patients with such foot ulcers go on to need an amputation [3].

Diabetes-related lower-extremity amputations rates are important indicators for the effectiveness of health care provided to diabetic patients including prevention and management of foot ulcers and to forecast the magnitude of the problem [4]. Previous studies have reported that early identification of people at high risk for foot problems and management of the risk factors could prevent lower extremity amputations and foot ulcerations [5, 6]. To this end, identifying the role of risk factors for diabetic foot ulceration will enable health providers to implement better prevention programs that could result in improved patient quality of life and, thus, reduce the economic burden for both the patient and the health care system [7]. Hence, the findings of the present study could help diabetologists recognize early, and manage diabetic foot, and thus reduce the limb amputation risk, and the cost that accompanies limb loss in this prevalent condition.

## Main text

### Ethical considerations

The study was conducted in accordance with the ethical standards of the institutional and national research committee and with the Helsinki Declaration and its later amendments. It was approved by the Medical and Bioethics local committee of Northern Border University. Participants were informed about the purpose of the study and data were collected after obtaining written consent from each patient. They were allowed to refuse or discontinue the participation at any time at request. Information was recorded anonymously and confidentiality and beneficence were assured throughout the study.

### Methods

A facility based matched prospective case–control study was conducted to collect data from a total of 200 consecutive Saudi T2DM patients (with/without DF; n = 100 for each group) presented during their routine visit to the diabetic center at the public Prince Hospital, Arar, Saudi Arabia from April 2018 up to May 2018. Data were collected using (1) interviewer administered structured questionnaire which was administered by the medical students for each diabetic patient and adopted by the local hospital protocols for diabetic foot, including the patient age, history of smoking, duration of DM, presence of hypertension and the current medication use, (2) observational and (3) chart analysis. The questioner reliability was previously checked by the hospital medical staff using coefficient alpha.

Body mass index [BMI; weight (kg) divided by height (m) squared (kg/m^2^)] and blood pressure (measured by an electronic vital signs monitor (SureSigns VS3, Philips Medical System, MA, USA) have been measured.

Permission was taken from the hospital administration to allow the researchers to revise the patients’ medical records archived in a specified diabetic center system (Medical plus). The nature of diabetic foot lesion and complications (i.e. ulcer, gangrene or amputation) [8], peripheral vascular pulses [9] and peripheral neurological status [10, 11], were retrieved from patients’ medical records. Diabetes glycemic parameters namely; fasting blood glucose (FBG; mmol/l) and hemoglobin A1c (HbA1c %) levels were collected from patients’ laboratory data according to their latest hospital visit.

#### Statistical analysis

Data analyses were conducted using Statistical Package for the Social Sciences (SPSS) software version 23 (IBM SPSS Inc, Chicago, IL, USA). Shapiro–Wilk test and Levene test were applied for data distribution and variance homogeneity check, respectively. Sample size of the study was calculated using G power software (http://www.gpower.hhu.de). Calculations showed that with the specified study design (case–control), and allowable error rates; alpha error = 0.5 with sample size 100 for each group can give 94% power with an effect size = 0.5. All the categorical variables were presented as number (percentage) and compared by Chi square (χ^2^) or Fisher’s exact tests where appropriate. All the continuous variables were presented as mean ± standard deviation and the Student̕ s t test was used for sub-group comparison. Univariate analysis was performed to identify factors associated with DF among T2DM. Bivariate correlation matrix using Spearman’s rank correlation analysis was applied to correlate between different parameters of the study. Multivariate analysis using the principal component analysis for data exploration was run to test the possibility of patients clustering according to the study variables. Significance was set at *P* < 0.05.

### Results

#### Baseline characteristics of the study population

**Fig. 1:**
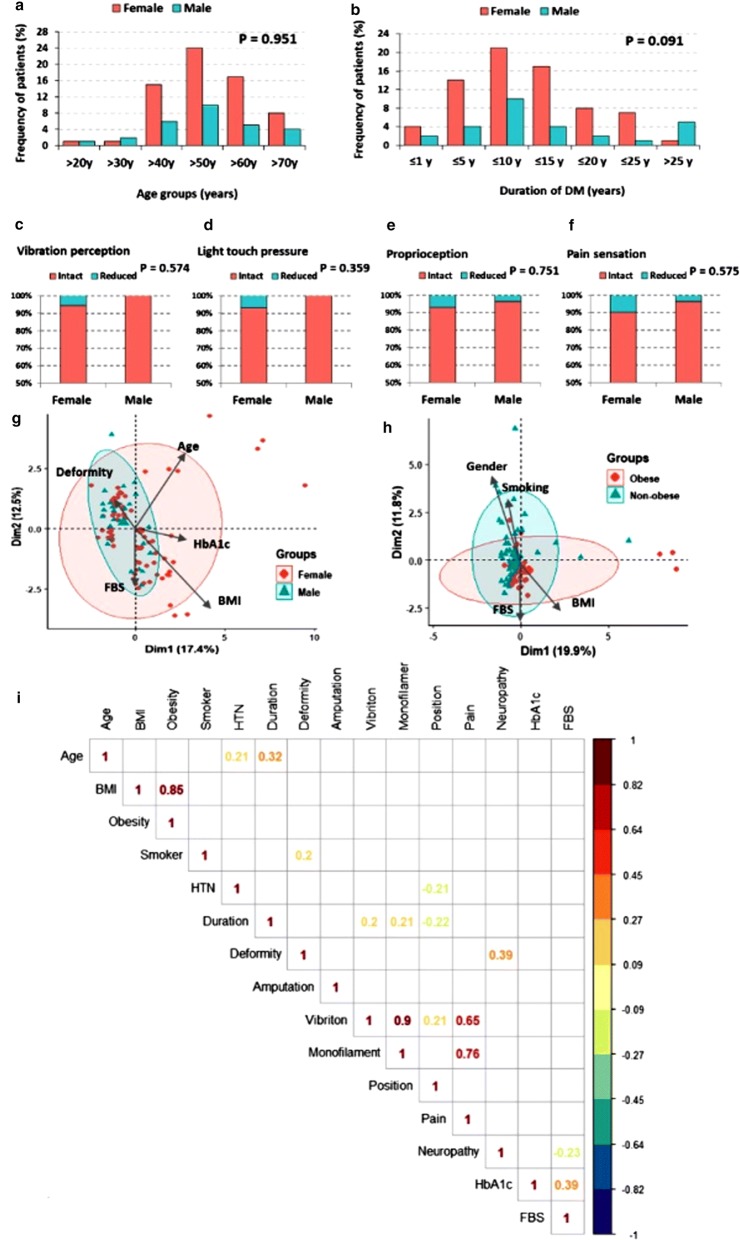
Clinical characteristics of diabetic foot ulcer patients. **a**, **b** Frequency of males and female patients according to their age group and duration of diabetes disease. **c**–**f** Frequency of some clinical features of T2DM with foot ulcer. **g**, **h** Multivariate analysis using Principal Component Analysis. Results were plotted on both axes and stratification was done by gender (**g**) and obesity (**h**). **i** Bivariate correlation matrix using Spearman’s rank correlation analysis, only significant values are shown. Chi square/Fisher’s exact tests were used for comparison between frequencies. *Dim* dimension, *BMI* body mass index, *HbA1c* glycated hemoglobin, *FBS* fasting blood sugar, *HTN* hypertension

Patients’ baseline characteristics are shown in Table 1 and Fig. 1a. The T2DM female patients were more prevalent in both studied groups. The mean age of participants, mean BMI, presence of obesity and current smoking status were similar in both T2DM with/without DF. Univariate analysis showed that higher frequency of older age, longer mean duration of diabetes, and higher mean HbA1c (%) levels were evident in T2DM with DF [OR (95% CI): 4.1 (2.3–7.4), 6.5 (4.9–9.3), 1.1 (1.05–1.3), respectively].

**Table 1:** Baseline characteristics of study population

	T2DM patients				T2DM patients with foot ulcer				*P* value^a^	OR (95% CI)
	Total	Female	Male	*P* value	Total	Female	Male	*P* value		
Total number	100	71	29		100	72	28			
Age (years)	56.4 ± 12.2	55.9 ± 11.8	57.1 ± 13.2	0.638	56.7 ± 12.2	56.5 ± 11.7	57.1 ± 13.4	0.798	0.862	
Age categories (years)										
≤ 50	66 (66.0)	48 (67.6)	18 (62.1)	0.080	32 (32.0)	23 (31.9)	9 (32.1)	0.985	< *0.0001*	*4.1 (2.3*–*7.4)*
> 50	34 (34.0)	23 (32.4)	11 (37.9)		68 (68.0)	49 (68.1)	19 (67.9)			
Body mass index										
(Kg/m^2^)	31.0 ± 6.1	31.8 ± 6.7	28.3 ± 4.0	*0.010*	30.0 ± 6.2	30.8 ± 6.9	28.1 ± 3.9	0.061	0.251	
DM duration (years)	13.4 ± 7.9	13.7 ± 6.4	13.2 ± 9.2	0.757	20.5 ± 7.6	19.9 ± 6.8	20.2 ± 9.1	0.857	< *0.0001*	*6.5 (4.9*–*9.3)*
Obesity										
Negative	68 (68.0)	48 (67.7)	20 (69.0)	0.894	59 (59.0)	39 (54.2)	20 (71.4)	0.174	0.187	1.5 (0.8–2.6)
Positive	32 (32.0)	23 (32.3)	9 (31.0)		41 (41.0)	33 (45.8)	8 (28.6)			
Smoking										
Negative	89 (89.0)	71 (100)	18 (62.1)	< *0.001*	94 (94.0)	72 (100)	22 (78.6)	< *0.001*	0.211	0.5 (0.2–1.5)
Positive	11 (11.0)	0 (0.0)	11 (37.9)		6 (6.0)	0 (0.0)	6 (21.4)			
Hypertension										
Negative	41 (41.0)	23 (32.3)	18 (62.1)	*0.006*	60 (60.0)	41 (56.9)	19 (67.9)	0.369	*0.007*	0.5 (0.–0.8)
Positive	59 (59.0)	48 (67.7)	11 (37.9)		40 (40.0)	31 (43.1)	9 (32.1)			
FBG (mmol/l)	9.0 ± 1.0	9.4 ± 0.9	8.7 ± 0.7	< *0.001*	9.2 ± 2.9	9.4 ± 3.0	8.51 ± 2.7	0.159	0.515	
HbA1c (%)	8.0 ± 0.4	8.3 ± 0.5	7.9 ± 0.2	< *0.001*	8.5 ± 1.5	8.5 ± 1.4	8.6 ± 1.7	0.653	*0.002*	1.1 (1.05–1.3)

Data are shown as mean ± SD or number (percentage). *Obesity* (*positive*) a body mass index of 30 kilograms divided by height in meters squared (kg/m^2^) or above, *Hypertension* (*positive*) history of treated hypertension or blood pressure ≥ 140/90 mmHg, *FBG* fasting blood glucose, *HbA1c* Glycated hemoglobin, *OR 95% CI* Odds ratio 95% confidence interval. ^a^*P* value was calculated using total T2DM with and without DF. Student’s t and Chi square/Fisher’s exact tests were used for comparison. Italic values indicate significance at *P *< 0.05

Compared to male participants, female patients had higher values of BMI in T2DM with/without DF (*P *= 0.010 and 0.061, respectively), but with negative history of smoking. Primary hypertension was reported in 40% and 59% of the patients with/without DF, respectively. Most of T2DM patients with DF (76%) were taking oral hypoglycemic drugs only or with insulin (7%). Around half (45%) of them had diabetes more than 10 years; 31% were of duration between 10 and 20 years, 12% and 2% were of duration more than 20 and 30 years, respectively, whereas 31% were of duration between 5–10 years and only 6 patients were newly diagnosed. The proportion of male and female patients with different durations of diabetes mellitus among T2DM with DF has illustrated in Fig. 1b. As an overall trend, more men had prolonged disease span ≥ 25 years long compared to women, unlike other age categories (P = 0.091). As can be seen, large sector of females experienced the disease from 5 to 15 years, whereas, high frequency of males were categorized either at the disease duration between 5 and 10 or ≥ 25 years.

#### Characteristics of diabetic foot among T2DM with DF

All patients except one female patient with amputation below the knee had a single ulcer disease. Lesions in less than three quarters were in the left foot, and most ulcers were in the forefoot region. Local examination showed dryness and fissured skin with fungal infection (3%), claw toe deformity (2%) or hammer toe (1%). All patients had intact ankle reflex and most of them had intact sensation. Diabetic foot patients received mainly treatment for the infection and pressure offloading. Although stratification analysis by sex revealed that more trend of higher frequency of impaired vibration perception, light touch pressure, proprioception and pain sensation prevalent in females than males (Fig. 1c–f), these trends were not significant. Multivariate analysis by principal component analysis for data exploration revealed unclear demarcation between patients of different sex and regarding obesity (Fig. 1g, h). The bivariate correlation matrix showed significant correlations between duration of disease and several diabetic foot manifestations like impaired vibration perception, pressure sensation by monofilament test, and impaired position sensation albeit they were weak correlations (r = 0.20, 0.21, and 0.22, respectively).

#### Biochemical analysis

Large percentage of patients (89%) had poor glycemic control (HbA1c ≥ 7.0%) with (mean ± SD); 8.5 ± 1.5 and 8.0 ± 0.4% in T2DM with/without DF, respectively. Female patients presented with higher levels of mean FBG and HbA1c levels than males in T2DM without DF (*P* < 0.001).

### Discussion

In light of the great burden of T2DM and its complications including the DF disease in Saudi Arabia where disease prevalence ranges from 11.4 to 29.7% [12, 13], the current study was conducted to identify the factors associated with DF in the researchers’ area “Northern Borders of Saudi Arabia”. DF disease is one of the major health problems that impairs the patient’s quality of life, entails high cost, and requires prolonged hospitalization [14]. Furthermore, most amputations begin with ulcers and can be prevented by good foot care and screening to assess the risk of foot complications [15].

The mean age of the study participants was about 56 (± 12.2) years with about 68% of them aged above 50 years. In univariate analysis, older age was associated with higher frequency of diabetic foot occurrence. This finding was comparable with Al-Rubeaan et al. [7] in their retrospective cohort study where they reported that “age ≥ 45 years is a risk factor for developing diabetic foot ulcers in Saudi population”. Similarly, in the study about the factors associated with amputation among DF ulcer patients in Saudi population, Musa and his colleagues [16], recently highlighted the “epidemic of metabolic syndrome around the world”, which particularly affects the Gulf region, being responsible for a younger age at the onset of the disease.

Contrary to the findings of some studies that found that male sex was predominant in DF patients [9, 17], female patients in the current T2DM cohorts were more prevalent. The influence of sex on DF disease was controversial [18]. Dinah and Vives found in their multicenter analysis, which included 248 T2DM patients with DF, that sex may imply a significant risk factor for the development of diabetic ulcers [19]. They argued that females could have a lower risk relative to males partly because of less severe neuropathy, increased joint movement, and lower foot pressure. However, once neuropathy or other risk factors for DF are present, both sexes have equal risk of DF development. The authors cannot exclude the impact of gender norms on women’s health in the local area. According to Aldosari [20], “women may find that health services are inaccessible or conditioned on certain cultural grounds or gender norms, such as restrictions imposed by the male guardianship system, women’s driving ban, gender segregation and religious norms affect access, quality and outcomes for women in Saudi Arabia”. These factors can also contribute to the more trend of higher frequency of DF-related manifestations observed in the current female patients than males. Large-scale studies are warranted to validate this issue.

The current study revealed that 41% and 32% of T2DM with/without DF were generally obese. Despite a number of studies shows a correlation between increased body weight and increased risk of foot ulcers [21, 22], due to a speculated effect of body weight on planter pressure, however, such correlation was not consistent [23].

Our univariate analysis also revealed that the duration of diabetes was a major contributory factor to the increased incidence of DF disorders (nearly by 6.5-fold). This finding was in line with the previous studies that support the association between prevalence of DF and duration of diabetes [24–27]. Also, it is worth noting that DF disorders are common among diabetic patients even at the early stage of diabetes or at the time they were diagnosed as T2DM [28]. Although the duration of diabetes is not a modifiable risk factor, it is of great importance for early identification and management of DF disorders as stated by Alzahrani et al. [9].

Higher frequency of the study patients (89%) were reported to have poor glycemic control that showed significant association with DF development. This finding was consistent with other studies that reported poor glycemic control as one of the main factors implicated in DF problems [29, 30]. Previous studies have also shown that HbA1c was a contributory factor for DF ulcer [19, 31, 32]. This may be due to hyperglycemia, which has been considered a risk factor for the development of DF ulcers because of its contribution toward the development of peripheral neuropathy and microvascular complications [25].

Taken together, this study identified that the older age, disease duration and poor glycemic control are significant risk factors related to DF development in the current T2DM population. Most of these factors are correctable or at least controllable with a large opportunity for early prevention and treatment, with the subsequent reduction in patients with DF and its devastating sequel of amputation.

## Limitations

-  This study is quantitative; it was better if qualitative approach was also employed, which is recommended in the future studies.
-  The adjustment for the other potential major contributing factors and other diabetic microangiopathic complications was not carried out in the current study.
- The authors did not consider some potential confounders in the occurrence of new foot ulceration such as health care provision level and patient behavioral factors like compliance with training on their foot care.

## Abbreviations

BMI: body mass index
DF: diabetic foot
FBG: fasting blood glucose
HbA1C: glycated hemoglobin
SPSS: Statistics Package for Social Science
T2DM: type 2 diabetes mellitus
